# Convection Enhanced Delivery in the Setting of High-Grade Gliomas

**DOI:** 10.3390/pharmaceutics13040561

**Published:** 2021-04-15

**Authors:** Chibueze D. Nwagwu, Amanda V. Immidisetti, Michael Y. Jiang, Oluwasegun Adeagbo, David C. Adamson, Anne-Marie Carbonell

**Affiliations:** 1 Emory University School of Medicine, Atlanta, GA 30322, USA; oadeagb@emory.edu; 2 Robert Wood Johnson Medical School, Rutgers University, New Brunswick, NJ 08901, USA; avi6@rwjms.rutgers.edu; 3 Imperial College School of Medicine, London SW7 2DD, UK; jiangmichael41@gmail.com; 4 Department of Neurosurgery, School of Medicine, Emory University, Atlanta, GA 30322, USA; cory.adamson@emory.edu; 5 Atlanta VA Healthcare System, Decatur, GA 30033, USA; 6 OncoSynergy, Inc., Stamford, CT 06902, USA; anne-marie@oncosynergy.com

**Keywords:** glioblastoma, high-grade glioma, refractory glioma, direct delivery, convection enhanced delivery, neuro-oncology, refractory glioblastoma, clinical trials

## Abstract

Development of effective treatments for high-grade glioma (HGG) is hampered by (1) the blood–brain barrier (BBB), (2) an infiltrative growth pattern, (3) rapid development of therapeutic resistance, and, in many cases, (4) dose-limiting toxicity due to systemic exposure. Convection-enhanced delivery (CED) has the potential to significantly limit systemic toxicity and increase therapeutic index by directly delivering homogenous drug concentrations to the site of disease. In this review, we present clinical experiences and preclinical developments of CED in the setting of high-grade gliomas.

## 1. Introduction

### 1.1. Glioblastoma

Glioblastoma (GBM) is the commonest malignant brain tumor of primary origin. Despite an annual incidence of less than 10 per 100,000 people worldwide, this aggressive and invasive glioma has a devastating prognosis with a current 1-year survival rate of 42.8% and 5-year survival rate at an abysmal 7.2% [1]. Often characterized as molecularly heterogenous tumors, the recent c-IMPACT-now consensus classified GBM as isocitrate dehydrogenase (IDH)-wild type WHO grade IV glioma with microvascular proliferation, necrotic lesions, or possessing > 1 of the following genetic alterations: TERT promoter mutation, EGFR gene amplification, +7/−10 chromosome copy number changes [2,3]. The current standard of care consists of maximally safe surgical resection with concurrent radiochemotherapy followed by at least 6 months of chemotherapy [4]. Complete resection of malignant tissue is limited by the highly invasive nature of this malignancy, thereby creating a reliance to chemotherapeutic options such as temozolomide, which have traditionally been delivered systemically. The restrictive blood–brain barrier (BBB) and toxicities from systemic delivery have limited therapeutic options.

### 1.2. Blood–Brain Barrier

An integral reason for the limited success of IV chemotherapy is the highly selective permeability of the BBB [5]. This filter typically only permits passage of lipophilic molecules smaller than 500 Da, hampering systemic delivery of chemotherapy and molecular-targeting constructs [6,7]. Thus, alternative delivery methods such as direct delivery have been explored. 

### 1.3. Direct Delivery

Direct delivery circumvents the aforementioned limitations posed by the BBB by allowing direct administration of BBB-impermeable drug substances into malignant tissues. This not only achieves a localized chemotherapeutic response, but also manipulates the stringent nature of the BBB to the clinician’s advantage by preventing drug substances from entering the systemic circulation and causing toxicity. These methods include implantation of chemotherapy-embedded biodegradable wafers, stereotactic injection, and convection-enhanced delivery (CED). Wafers deliver high drug concentrations into tumor resection cavities, but effects are limited to a few millimeters from the implantation site [8,9,10]. Given that the overwhelming majority of recurrences occur within 2 cm of the tumor border, additional methods are warranted to provide more complete coverage of this region of the tumor-infiltrated brain [11,12,13,14]. Stereotactic injection relies heavily on conventional diffusion of drug products, which considerably hampers intended infusion parameters and pharmacokinetics due to a steep drop in drug concentration. As a result, higher concentrations must be infused during stereotactic injection to overcome the limitations of conventional diffusion posed by physiological brain morphology [15,16]. CED obviates these shortcomings by leveraging pressure differences to deliver homogenous drug concentrations to a greater volume of targeted tumor areas [17,18,19].

### 1.4. Convection Enhanced Delivery

Developed in the 1990s by Bobo et al., CED is a unique infusion technique whereby pressure-driven catheters are utilized to drive infusate directly into a localized area of parenchymal tissue within the central nervous system (CNS) [20]. This approach focuses on establishing a pressure rather than concentration gradient to enhance therapeutic delivery. Traditional infusion techniques rely on diffusion primarily, necessitating large concentration gradients to maximize molar flow as per Fick’s law. Alternatively, generating a pressure gradient allows infusate to be distributed via bulk flow, where molar velocity is directly proportional to the pressure gradient as per Darcy’s law. Clinical application involves stereotactically siting catheters via burr holes into malignant CNS tissues under magnetic resonance imaging (MRI) guidance. Catheters are then connected to extra-cranial infusion pumps to establish a pressure gradient at the tips, distributing infusate into the tumor bulk by convective transport regardless of molecular size. Thus, therapeutic agents can penetrate tissues in the order of centimeters within a pseudo-spherical distribution from the catheter tip as opposed to only a few millimeters in diffusion-dependent infusion modalities [17]. Due to the steep pressure gradient generated, CED also increases peri-tumoral interstitial fluid flow (IFF), which has been associated with altered molecular transport and extracellular matrix (ECM) rearrangement. Computational modeling of IFF has thus been explored with varying success in light of its notable implications in predicting molecular transport and maximizing therapeutic delivery [21]. 

**Figure 1:**
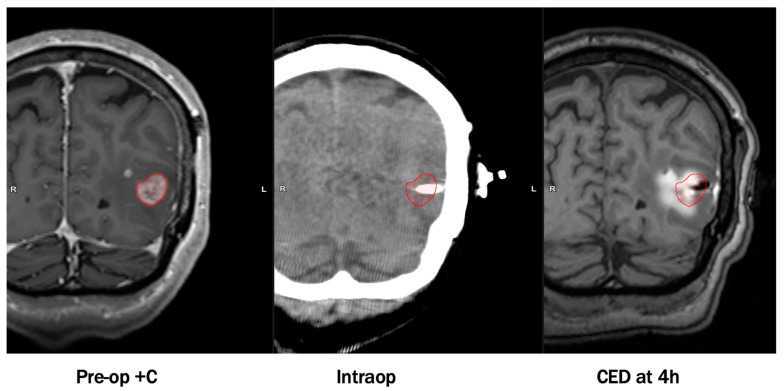
An example of an infusion performed by convection-enhanced delivery (CED) visualized on magnetic resonance imaging (MRI) with co-convected contrast. Images were obtained with permission from Dr. Michael Vogelbaum and Moffitt Cancer Center.

By avoiding systemic exposure, the maximum tolerated dose is greater if delivered by CED compared to systemic delivery [5,8,22]. In spite of this, CED usually does not require as high a concentration as stereotactic injection to attain therapeutic levels in affected parenchyma [17]. However, this procedure is not without its caveats. The most common limitations undermining previous CED trials consist of infusate reflux around the perimeter of catheters, suboptimal catheter placement, use of non-specialized catheters, an inability to trace drug substances, and risks relating to placing catheters (~1% risk of hemorrhage/catheter placed; ~5% risk of infection). Recent modifications have addressed these issues and led to considerable improvements in pharmacokinetic and infusion parameters. Notable adaptations involve co-administering drug products with contrast-enhancing agents, utilizing computer software to optimize catheter placement, and design of specialized CED catheters to prevent infusate reflux and leakage. An example of an infusion performed by CED visualized on MRI with co-convected contrast is shown in Figure 1. 

CED trial results have provided further insight into the efficacy and adverse effects possibly associated with this delivery method. Here, we also present a comprehensive review of clinical trial experiences with CED and recent preclinical developments in consideration for this drug delivery method. 

## 2. Clinical Experiences with Convection Enhanced Delivery

Convection enhanced delivery has been explored clinically with the use of a variety of therapeutic candidates including cytotoxic agents, recombinant toxins, viral vectors, and liposomally encapsulated particles. CED trials vary in therapeutic agents delivered, catheter design, number of catheters, infusion parameters, and methods to assess drug distribution. When assessing distribution of infusate across various trials, it is critical to consider the type of catheter used—some trials employ “off-the-shelf” catheters, which are repurposed catheters originally produced for other indications, and other trials use catheters that are specifically designed to optimize CED. This constellation of variables often complicates the ease of comparison between trials. It is prudent to group clinical experiences by the therapeutic agent delivered, as this can yield insights both into the efficacy of the therapeutic agent, efficiency of drug delivery, and elucidate the etiology of any shared adverse events that are exclusive to a group of trials using a similar agent. A summary of notable trials grouped by therapeutic agent is included in Table 1.

### 2.1. Paclitaxel

Paclitaxel (Taxol) is an antineoplastic agent that promotes the stabilization of microtubules to effectively cause mitotic arrest of neoplastic cells [42]. It has been explored as a treatment in a wide range of indications in oncology and is on the World Health Organization’s list of essential medicines (WHO list) [43]. 

A Phase I/II study was conducted in which fifteen patients with recurrent glioblastoma were given up to three intratumoral infusions of paclitaxel via CED [23]. In this trial, the distal portions of silicone ventriculoperitoneal shunt catheters were cut to produce a single opening at the tip. Following confirmation of catheter placement on MRI, infusion was initiated 0.3 mL/h in all groups. For the first cycle of infusions, the first three patients were started at a dose of 7.2 mg paclitaxel in 6 mL of normal saline per day (1.2 mg/mL). Subsequent patients received 3.6 mg paclitaxel in 6.6 mL of normal saline per day (0.55 mg/mL). The syringe and paclitaxel were replaced every 12 h to mitigate concern of precipitation or crystallization. Among these patients, 20 cycles of paclitaxel were administered in total: 11 for five days, two for four days, one for three days, and six for two days. Six patients’ treatments were discontinued due to complications, including fluid penetration into a cystic area and accidental catheter removal. Adverse events included chemical meningitis (*n* = 6), infection including meningitis and subdural empyema (*n* = 3), and transient neurological deterioration (*n* = 4), which was attributed to edema. Three patients required resection of necrotic tumor due to peritumoral edema. Median survival was 7.5 months, with two patients alive at 12 and 24 months at the time of publication. Antitumor response was noted in 11 out of 15 patients (14 of the 20 cycles administered), and there was a 73% response rate. A limitation of this study is the small sample size (*n* = 15).

In this trial, catheter tips were initially placed within the center of the contrast enhancing tumor mass. With this technique, the investigators observed leakage of the infusate into the subarachnoid space, ventricles, and cavities in tumors with widespread necrosis. In subsequent infusions, catheters were placed into enhancing tumors as distant as possible from the ventricles, cysts, or necrotic cavities. When diffusion-weighted imaging (DWI) performed before and during the infusion was compared, convection of the infusate appeared to stop when it reached a cystic area. This was associated with clinical symptoms consistent with chemical meningitis and lack of tumor response, supporting the possibility that the infusate was delivered into a CSF compartment rather than into tumor tissue. The authors also note that paclitaxel is prepared with a vehicle which may have irritated the leptomeninges upon contact and be responsible for the dose-dependent chemical meningitis that was observed. An interesting observation noted in this trial is that simple diffusion of infusate continued to occur briefly after CED was discontinued. Although simple diffusion typically accounts for a relatively small distance, since the starting point of the simple diffusion was relatively large (the entire volume of distribution of the treated area), even a short distance of simple diffusion was significant in some patients. 

Recommendations from this trial regarding concentration, infusion rate, and surgical strategy were implemented in a subsequent Phase I/II trial of intratumoral CED of paclitaxel in eight patients with recurrent glioblastoma [24]. In this trial, off-the-shelf catheters with an inner diameter 1.5 mm and outer diameter 3 mm were trimmed distally to create a single opening at the catheter tip. These catheters were again placed into the area of maximum enhancement; however, this protocol required that catheter tips were placed at least 15 mm from the cortex and 5 mm from the ventricles. If two catheters were used, they were separated by at least 20 mm. This trial consisted of three groups that all received 26 mL infusions under varying parameters. The first group of patients in this trial were treated at 0.5 mg/mL paclitaxel infused at a rate of 0.3 mL/h for 120 h. In these patients, significant backflow and efflux of the infusate out of the burr hole through which the catheter was placed was observed. Adverse events in this group included severe skin necrosis and impaired wound healing. To mitigate the backflow of infusate out of the cranium, in group 2, the burr hole through which the catheter was inserted was sealed with bone wax prior to the infusion and re-sealed upon removal of the catheter after the infusion. Remarkably, a significant increase in Vd was noted between group 1 (unsealed burr hole) and group 2 (sealed) from 12.8 to 22.9 cm^3^. In fact, the Vd in group 2 increased to such a degree that it exceeded tumor boundaries and was thought to cause neurological deficits. Since the study goal was to treat infiltrative disease, the same volume of infusion was maintained for group 3; however, the concentration of paclitaxel was reduced to 0.25 mg/mL in an effort to reduce neurotoxicity. In this group, the Vd was 20.5 cm^3^. No adverse events were noted in this group. One patient had a markedly low Vd of 7.8 cm^3^, which was thought to be due to the placement of a catheter only 3 mm from a ventricular space. Two patients in this trial had comparatively large tumors, which were treated with two separate catheters each with a reduced infusion rate of 0.2 mL/h per catheter. Survival ranged from 4 to 15 months. 

Arguably the most critical finding of this study was the significant increase in Vd of infusate upon sealing the burr hole through which the catheter was inserted. The authors posit that this phenomenon was not due solely to the closure of the hole and likely was influenced by the implication of changing the intracranial pressure, possibly eliminating paths of least resistance in which infusate may accumulate. It is also thought that sealing the burr hole may have provided additional stability to the catheter apparatus and eliminated motion artifact that could adversely influence the Vd. Finally, the authors note that paclitaxel is suspended in an oily vehicle that may not closely follow the behavior modeled for hydrophilic solutions. 

A consideration for future studies was that DWI was not accurate enough to measure Vd, as the authors state that it was unclear whether enhancement was due to fluid being pushed away by infusate or due to alterations in diffusion secondary to treatment-related apoptosis. 

A third study delivered intratumoral paclitaxel via CED to eight patients with recurrent glioblastoma [25]. This was the first study to use metabolic imaging to monitor effects of CED. In this trial, the tip of a silicone catheter was placed into the geometric center of each tumor and patients underwent 120 h infusions at a flow rate of 0.3 mL/h. The first group of four patients received a concentration of 0.5 mg/mL. Due to extensive skin necrosis and edema at this dose level, the concentration for the subsequent group was reduced to 0.25 mg/mL. In addition to MRI and DWI as described in the protocol, *O*-(2-[18F]fluoroethyl)-l-tyrosine (FET) positron emission tomography (PET) were obtained before CED, 4 weeks after CED, and at 3 month intervals thereafter. When considered together, areas of paclitaxel distribution on DWI correlated with lower FET uptake on subsequent PET scans and areas of poor convection on DWI were associated with high FET uptake. These findings highlight the potential for the use of metabolic imaging in long-term monitoring of agents delivered by CED; however, this requires validation in future studies. A greater degree of convection was observed along white matter tracts relative to grey matter, consistent with findings from Voges et al. [37]. This is thought to be due to a path of least resistance along the fibers of white matter tracts, through which infusate flows more freely than through grey matter. 

Adverse events included worsening neurological symptoms in six patients (irreversible in one), as well as poor wound healing due to leakage of paclitaxel through the burr hole in two patients, consistent with findings from Tanner et al. [24]. Clinical outcomes in this trial were variable. Two patients treated at the higher dose level rapidly deteriorated and died 4 and 5 months after treatment. Both patients had recurrent tumors with large necrotic areas, which may have contributed to further necrosis and edema following treatment. Six patients had temporarily stable disease and five patients developed recurrence at 3–13 months. All recurrences in these patients were at sites distant from the area where CED was performed. 

### 2.2. Topotecan 

Experiences with topotecan, a cytotoxic inhibitor of topoisomerase I, highlight the significance of locoregional delivery. Despite significant therapeutic effect when delivered intravenously, CED of topotecan in a preclinical rat model demonstrated efficacy and prolonged survival, paving the way for clinical trials [44,45]. In a phase Ib study of 16 patients with recurrent malignant glioma, two CSF-peritoneal catheters were placed either directly into the tumor or into the surrounding tumor infiltrated brain so as to maximize coverage [26]. Patients were enrolled into five dose levels at 0.02 to 0.133 mg/mL. Each patient received 40 mL of infusate at the assigned concentration at a flow rate of 200 μL/h per catheter for 100 h. Tumor volume was subsequently assessed by contrast enhancement on MRI. Two patients at the highest dose level (1.33 mg/mL) experienced toxicities including right parietal syndrome, dysmetria, hemineglect, and upper extremity weakness; some of which were transient and caused the infusion to be terminated early in one patient. The maximum tolerated dose (MTD) was 0.1 mg/mL. The total response rate to a single infusion was 69%. Median OS was 60 weeks and PFS at 6 months was 44%. 

A subsequent trial investigated the co-infusion of topotecan with gadolinium-diethylene-triamine-pentaacetic acid (Gd-DTPA), which enabled direct visualization of infusate. This trail was unique in its use of the Cleveland Multiport Catheter (CMC), which was specifically designed for CED [27]. Notable features include four independent-lumen polytetrafluoroethylene microcatheters within each barium-impregnated silicone central shaft. The microcatheters remain withdrawn within the central shaft during catheter placement until the stylet is removed, which causes the microcatheters to deploy radially. Three patients (two with glioblastoma and one with anaplastic astrocytoma) were enrolled. Two CMCs were placed per patient, both into the tumor and into the tumor-infiltrated brain. Each patient was infused with a total volume of 38 mL of 0.067 mg/mL topotecan and 5 mm Gd-DTPA at a rate of 0.396 μL/h (6.6 μL/min) over 96 h. This protocol was well tolerated and there were no instances of infection or hemorrhage. Notably, this study found that Vd of infusate was a function of catheter location, as Vd in the non-enhancing tumor-infiltrated brain was significantly higher than the Vd of intratumoral infusion (19.4 vs. 1.7 cm^3^). The authors posit that this disparity in Vd is likely due to areas of disrupted BBB in the contrast-enhancing tumor which facilitate greater efflux of infusate. The authors note that since Gd-DTPA and topotecan are closely matched in size, the Vd of tracer in this study is likely a close surrogate for the Vd of topotecan, however, other molecular properties may cause variability between the two. The results from the first three patients suggested that these infusion parameters were insufficient to overcome the efflux associated with contrast-enhancing tumor, thus, this trial was terminated. 

### 2.3. Conjugated Toxins

Recombinant toxins have been used in several CED trials. This pharmacotherapeutic approach involves utilizing a cytotoxic agent (commonly isolated from bacteria) and introducing selectivity by way of conjugation with a ligand whose cognate receptor is highly expressed in the target cell type [46]. Bacterial toxins utilized in this approach include Diphtheria toxin (a component of Tf-CRM-107) and Pseudomonas exotoxin (a component of TP-38, NBI-3001, and IL13-PE38QQR). 

#### 2.3.1. Tf-CRM107

Tf-CRM107 is a conjugate that contains a mutant Diphtheria toxin (CRM107) and human transferrin, which binds the transferrin receptors expressed in glioma cell lines [47]. An initial phase I trial was completed in 18 patients, who received concentrations of 0.1–3.2 μg/mL Tf-CRM107. Infusion was performed using one to three off-the-shelf CSF-peritoneal catheters that were repurposed for CED. Total volume delivered ranged from 5 to 180 mL at flow rates ranging from 0.5 to 10 μL/min. Adverse events included transient worsening of neurological deficits (thought to be secondary to increased cerebral edema), and peritumoral cerebral injury, which occurred in three out of four patients who experienced seizures. A concentration- and dose-dependent response was observed. Outcomes were significant for a greater than 50% decrease in tumor volume (observed in 60% of patients) and two complete responses. These findings prompted a subsequent phase II trial, in which 44 patients with recurrent glioblastoma or anaplastic astrocytoma were enrolled. Two catheters were placed into each patient and Tf-CRM107 was infused at a concentration of 0.67 μg/mL at 0.2 mL/h per catheter [29]. The infusion was continued for 4–5 days or until 40 mL of infusate was delivered. Thirty-one patients received a second infusion 4–10 weeks after the first. Median and mean survival were 37 and 45 weeks, respectively. Adverse events included symptomatic progressive edema in eight patients (14%) and seizures in three patients, which were controlled with anti-convulsants. The authors note that there were MRI changes that suggested microvascular injury and venous thrombosis, which may correlate with greater transferrin receptors on endothelial cells. Thus, toxicities may be due to the drug itself rather than to the infusion and the transferrin receptor may not be a specific enough target for locoregional therapy. 

#### 2.3.2. NB3001 (IL-4 PE, IL-4(38-37)-PE38KDEL)

NB3001 is a recombinant fusion protein that contains components of human interleukin-4 (IL-4) and a truncated form of Pseudomonas exotoxin. A phase I study was conducted in 31 patients with recurrent malignant glioma [30]. Up to three catheters were placed intratumorally and infusion was administered over 96 ± 4 h. Patients were enrolled into four dose groups at three concentrations (6, 9, and 15 μg/mL) and received 40 mL of infusate. The fourth dose group received 100 mL infusate at a concentration of 9 μg/mL. Drug-related grade 3 and 4 toxicities were seen in 39% of patients, the most common of which were seizures, headache, weakness, and cerebral edema. Serious adverse events included pulmonary embolism (12%), somnolence (10%), weakness (10%), coma (10%), and post-operative wound infection (10%). Median overall survival was 8.2 months (5.8 months for glioblastoma). An increase in contrast enhancement was observed 4 weeks post-infusion and decreased gradually over the next several weeks. Although the etiology is unclear, it may be associated with tumor necrosis, edema, inflammation, and the breakdown of the BBB. In future studies, the authors propose a reduction in concentration over an increased volume to increase Vd (thereby increasing tumor coverage) to possibly improve the safety profile of this therapy. Additionally, they note that the majority of adverse events were due to increased intracranial pressure secondary to cerebral edema, thus subsequent trials that incorporate tumor resection may circumvent these complications. 

#### 2.3.3. TP-38

TP-38 is a chimeric protein that contains a fragment of Pseudomonas exotoxin and TGF-α, which confers specificity by binding epidermal growth factor receptor (EGFR), a molecule that is over-expressed in malignant gliomas [48]. A phase I trial was performed in which 20 patients with recurrent malignant glioma received TP-38 at three dose levels (25, 50, and 100 ng/mL) [31]. Two barium-impregnated off-the-shelf catheters that were repurposed for CED in this trial were placed in each patient to target either residual contrast-enhancing tumor or deep white matter surrounding previously resected tumor. All three concentration levels received a total volume of 40 mL over 50 h at a rate of 0.4 mL/h from each catheter. Adverse events included hemiparesis, fatigue, subdural hygroma, and one case of hemorrhage after catheter placement. Median survival was 28 weeks. A notable feature of this trial was that the last eight patients in this trial were co-infused with ^123^I radiolabeled albumin, which functioned as a tracer to allow for a surrogate measurement of infusate distribution. Visualization with single-photon emission computed tomography (SPECT) showed that the desired distribution was not achieved and halted dose escalation. Out of the eight patients who underwent SPECT imagine, five of them showed significant infusate leaks into the subarachnoid and CSF spaces, which hindered intraparenchymal distribution. The authors note that this may have been due to a catheter trajectory that perforated a deep pial or ependymal border, which would produce a path of least resistance for flow away from interstitial space. Additionally, the “off-the-shelf” ventricular catheter used in this trial had multiple perforations from the catheter tip to 17 mm proximally. The authors noted that this feature may have contributed to leaks into the CSF compartment, as infusate could exit the catheter more proximally through the perforations. In one case, the catheter trajectory crossed the Sylvian fissure and the most proximal port (located 17 mm from the tip) communicated with the subarachnoid space, which allowed infusate to leak into the CSF space between the frontal and temporal lobes. Although adequate distribution was achieved in some cases (a 4.1 cm distribution of at least 10% of the infused concentration was seen in one patient), only 3 out of 16 catheters produced adequate intraparenchymal distribution. These findings highlight the need to explore more strict catheter placement guidelines and potential for ineffective infusions when using multipurpose or “off-the-shelf” catheters that are not specifically designed for CED. 

#### 2.3.4. IL13–PE38QQR (Citredekin Besudotox)

IL13–PE38QQR is a recombinant chimeric cytotoxin that contains IL-13 and a fragment of Pseudomonas exotoxin. This agent has been widely explored in CED clinical trials. A phase 1 study enrolled 14 patients with malignant glioma into two groups [49]. The first received IL13-PE38QQR an intratumoral infusion, underwent tumor resection, and received an intraparenchymal infusion post-resection (treat-resect-treat study design). The second group only received an intraparenchymal infusion after resection only (resect-treat). Intratumoral infusions consisted of 0.35–2 μg/mL of IL13-PE38QQR delivered through a single catheter. A total volume of 36 mL were delivered over two days. The intraparenchymal infusions consisted of 0.5–1 μg/mL delivered through two to three catheters. A total volume of 72 mL was infused over four days. To avoid infusion into ventricles or the subarachnoid space as seen in earlier CED trials, catheters were placed around the resection cavity through separate small corticotomies rather than through the resection cavity itself. This study examined serial neuroradiographic changes in a cohort of patients within a larger ongoing trial to better characterize features due to procedure, infusion, drug effect, or tumor progression. Repeat biopsy is the gold standard; however, due to its invasive nature, it was performed in only 21% of patients in this study. This group developed a scale to grade likely treatment-related changes on the MRI in an attempt to differentiate them from tumor progression. Where treatment-related contrast enhancement was suspected, 71% of patients underwent further biological imaging (MR spectroscopy, MR cerebral blood volume studies, or FDG-PET). FDG-PET showed increased metabolic activity, MR perfusion revealed increased blood flow, and MR spectroscopy tended to be equivocal. MR changes observed along the catheter tracks and not associated with changes elsewhere were considered to be treatment related. Patients receiving 0.5 μg/mL or less of IL13-PE38QQR were found to have imaging changes that peaked around four weeks after therapy and generally became apparent by eight weeks. Patients in the highest dose group for intraparenchymal infusion following resection (1 μg/mL of IL13-PE38QQR) were found to have imaging changes that were progressive and predicative of neurological deterioration. This study elucidated post-CED treatment-related changes that could be used to better distinguish between treatment related changes and tumor recurrence in future CED trials. 

A phase I study of IL13-PE38QQR was performed in 22 patients with newly diagnosed malignant glioma. Patients were enrolled into two dose levels of 0.25 and 0.5 μg/mL of IL13-PE38QQR [50]. Following gross total resection, two to four barium-impregnated silicone catheters were placed, and the infusion was conducted at a fixed rate of 0.75 mL/h for 96 h. This was followed by external beam radiation therapy with or without temozolomide. Adverse events included fatigue, gait disturbance, nystagmus, and confusion. DLTs included a grade 4 seizure and grade 3 aphasia and confusion. No systemic toxicity was noted. Eleven out of 22 patients remained alive at the median follow up of 44 weeks and survival ranged from 5 to 113 weeks. 

A group of three phase I studies of IL13-PE38QQR treated 51 patients at six study sites [33]. In these studies, patients either underwent intratumoral infusion, resection, and subsequent intraparenchymal infusion (treat-resect-treat) or underwent tumor resection followed by a single intraparenchymal infusion (resect-treat). Intratumoral infusion was delivered through up to two catheters placed into the solid tumor component and infused doses of 0.25–2 μg/mL of IL13-PE38QQR. Infusion was conducted at a total rate of 0.4 or 0.54 mL/hour for 96 h and delivered 19.2–51.8 mL of infusate. Intraparenchymal infusion was delivered through up to three catheters which were placed in areas thought to be at risk for residual infiltrating disease. Infusion was conducted at a fixed rate of 0.75 mL/h from a period of 96 h up to six days. A total volume of 72–108 mL of infusate was delivered. This trial implemented three symptomatic windows by which to assess adverse events and attribute them to an underlying cause. Adverse events that occurred in the first window (from surgery to CED) were likely due to the surgical procedure. Events during the second window (from CED to 1 week after completion) were likely due to mass effect from the infusion, and events in the third window (2–10 weeks after treatment) were likely due to the drug’s effect on the brain. Most adverse events occurred during the second and third windows. Most common adverse events in the first window (pre-CED) included headache, hemiparesis, sensory disturbance, facial paresis, and limb ataxia. The second window (peri-CED) revealed headache, sensory disturbance, pyrexia, and memory impairment. Finally, the third window (post-CED) showed headache, aphasia, speech disorder, gait disorder, and asthenia. Adverse events in the second window were reduced when corticosteroid guidelines were implemented, which may have reduced the mass effect of infusate. The maximum tolerated infusion was 72 mL over the course of 96 h at 0.75 mL/h. 

In a separate analysis of catheter placement, a subset of six patients in one of the phase I studies in this series received co-infusion of human serum albumin radiolabeled with ^123^I (^123^I-HAS), a tracer used to visualize the infusate [51]. This analysis found that intraparenchymal distribution varied based on catheter positioning and only 10 out of 19 catheters that were placed achieved adequate distribution. Consistent with prior studies, leakage into the CSF compartment occurred when the catheter trajectory crossed a deep sulcus or if the catheter tip violated pial or ependymal surfaces. A major finding of this study was to clarify optimal catheter positioning, which was defined as depth ≥ 25 mm from brain surface, sulcus, or resection cavity, tip distance ≥ 10 mm from ependymal/pial surfaces, and tip distance ≥ 5 mm from resection cavity walls. Among 24 patients with glioblastoma, there was a statistically significant difference in survival among patients with two or more optimally placed catheters versus those without (55.6 weeks versus 37.4 weeks). Catheter depth was found to be a crucial consideration in placement. Deeply positioned catheters greater than 20 mm from all surfaces and sulci provided the best Vds. Additionally, these authors note that timing of catheter placement appeared to influence positioning, as immediate placement following resection was based on pre-operative MRI, which quickly becomes less accurate due to brain shift during resection. Post-operative edema may further displace catheters from their intended location. Based upon these findings, deferred placement using post-operative imaging may be indicated for more accurate catheter placement. 

A large phase 3 multicenter trial (PRECISE) enrolled 296 patients to receive either IL13-PE38QQR infusion or carmustine (Gliadel) wafer implant following tumor resection. In the experimental arm, 0.5 μg/mL IL13-PE38QQR was infused at a rate of 0.75 mL/h over 96 h through two to four intraparenchymal catheters. Catheter placement took place 2–7 days post-resection. Both arms had similar safety profiles and the occurrence of ≥ grade 3 AEs was not significantly different between the two groups except for pulmonary embolism, which was thought to be a consequence of the extended hospital stay in the CED group. The median survival was 36.4 weeks in the infusion study arm and 35.3 weeks in the carmustine wafer arm. The study endpoint, 50% improvement in median survival in the experimental arm, was not met. Despite rigorous investigator training in preparation for this trial, which included on-site training, off-site training, and four mock placements, only 68% of catheters were found to be positioned according to protocol guidelines. Subsequent analyses found that only 53% of patients in this trial had catheters with adequate positioning scores [52]. Furthermore, only 285 out of 572 catheters placed (49.8%) met positioning criteria. In fact, the majority of catheters placed in this trial failed to meet criteria. The estimated coverage of infusion was also low, and only 20.1% of the targeted 2 cm penumbra surrounding the resection cavity was covered [53]. These findings suggest that the failure to meet the efficacy endpoint in the PRECISE trial may have been due to catheter placement and infusion coverage rather than to the agent itself. This group also retrospectively tested a software algorithm using MR diffusion tensor imaging to predict drug distribution. The images from patients who were co-infused with the tracer 123I-HSA were compared with distribution simulations provided by the software. This was found to have a sensitivity of 71.4% and specificity of 100% for identifying catheter trajectories that failed to deliver the drug adequately. Concordance between tracer distribution and software simulation was 65.7%, and this was found to be clinically useful in 84.6% of placed catheters [54]. If further developed and validated, programs such as this have the potential to be valuable tools in pre-catheter placement planning. 

### 2.4. Viruses

#### 2.4.1. Reovirus

A phase I trial of reovirus (REOLYSIN), an oncolytic RNA virus, was conducted in 15 patients with recurrent malignant glioma [35]. This was the first attempted use of CED in direct delivery of a virus in patients with brain tumors. In this trial, reovirus was dosed at 1 × 10^8^ to 1 × 10^10^ tissue culture infectious dose 50 (TCID50). The first six patients were placed with two CED catheters each; however, intermittent clogging was noted in a majority of these patients and the protocol was amended to allow for up to four catheters to be placed. Catheters were placed at least 2 cm from the cortical surface and 1 cm from ventricles, sulci, and prior resection cavities. The infusion was delivered at a total rate of 400 μL/hour over a period of 72 h. There were no DLTs and a MTD was not identified. One grade 3 adverse event (convulsion) was deemed as being possibly related to treatment. There were no grade 3 or 4 adverse events that were considered “probably” or “definitely” related to treatment. Median survival was 140 days (range 97–989) and median time to progression was 61 days (range 97–989). Although a limitation of this study was the lack of tagged virus imaging, the authors note that pre- and post-infusion MRIs support the possibility that a larger Vd of the virus was achieved through the use of CED over injection. Additionally, despite the fact that a catheter specifically designed for CED was used in this study, it would be prudent to further investigate the reason for clogging, as this was a widely encountered complication in the first cohort of patients. 

#### 2.4.2. PVSRIPO

PVSRIPO, a recombinant poliovirus-rhinovirus that binds upregulated CD155 in neoplastic cells, was explored in a phase I trial in 61 patients with recurrent glioblastoma [36]. Patients received intratumoral infusions at one of seven dose levels (10^7^ to 10^10^ TCID50). The volume was held constant at 3.2 mL across all concentrations. A single catheter was placed at the time of resection and infusion took place over 6.5 h at a rate of 500 μL/h. One patient who received 10^10^ TCID50 experienced a DLT of grade 4 intracranial hemorrhage upon catheter removal, which prompted dose de-escalation. Common adverse events included headache, pyramidal tract syndrome (hemiparesis), seizure, dysphasia, and cognitive disturbance. Median overall survival was 12.5 months (95% CI, 9.9–15.2), which was an improvement over the historical control group which had a survival of 11.3 months (95% CI, 9.8–12.5). The authors note that although complete and partial responses were easily identifiable on imaging, assessment of tumor progression continued to be a challenge. 

#### 2.4.3. Delta-24-RGD

Delta-24-RGD is a conditionally replication-competent adenovirus that was investigated in a phase I study in 13 patients with recurrent glioblastoma. In an analysis published by van Putten et al., a test infusion of Gd-DTPA was conducted and studied to detect catheter dysfunction [55]. Up to four silicone ventricular catheters were placed either intra or peritumorally and 1 mm Gd-DTPA was infused at 0.2–0.3 mL/h for 6 h. At the conclusion of the infusion, T1-weighted (without contrast), T2-weighted, diffusion tensor, and FLAIR MRIs were obtained to assess for possible infusate leakage into the subarachnoid or ventricular spaces. There was one instance in which a catheter was unable to be placed due to persistent bleeding at the catheter entry point. A total of 51 catheters were analyzed—13 were placed peritumorally and 37 were placed intratumorally. Despite study guidelines that were designed to avoid leakage, 17 out of 50 catheters (34%) exhibited leakage into CSF. The most frequent cause of leakage was due to the intersection of catheter trajectory with a sulcus. In cases where leakage was detected, the mean distance from catheter tip to the nearest surface was 2.4 mm (range 0–5.4). In eight cases, leakage was not detected on T1-weighted imaging but was detected on FLAIR sequence. As expected, Vd/Vi ratio was larger in catheters which were not leaking. One of the most significant findings in this study was that intratumoral catheters had a significantly lower distribution of Gd-DTPA than peritumorally placed catheters. It was observed that infusate tended to pool in the necrotic portions of the tumor or leak through the enhancing portions of the tumor. These findings will likely guide future experiences in CED clinical trials, as certain tumors with large necrotic areas may not be ideal for intratumoral CED given the possibility of pooling.

### 2.5. Liposomes 

Liposomes are structures formed by phospholipid amphiphiles and cholesterol to encompass an aqueous internal domain and have been explored in CED trials [56]. Advantages of this approach include their relatively small size, ability to gain entry into cells by being phagocytosed or endocytosed, possibility to extend drug half-life, lack of immunogencity, and long-term stability at room temperature [37,56]. 

#### 2.5.1. LIPO-HSV-1-tk

LIPO-HSV-1-tk is a liposomal vector that contains the herpesvirus thymidine kinase (tk) gene [57]. Upon delivery of this gene, cells become sensitized to ganciclovir, a nucleoside analog that is clinically used as an antiviral. In a phase I/II study in eight patients with recurrent glioblastoma, LIPO-HSV-1-tk was infused intratumorally and followed with systemic ganciclovir [37]. Up to two silicon catheters were placed into each tumor and infused at flow rates from 0.025 to 0.6 mL/h to deliver a volume of 3.5 mL/catheter over 2 h. A notable feature of this trial was that two days before the planned infusion of LIPO-HSV-1-tk, six out of eight patients received a 3.5 mL per catheter test infusion containing 0.004 mmol Gd-DTPA. MR was done at 12, 14, and 16 h following this infusion. Gd-DTPA distribution was considered as a surrogate for the efficacy of CED. Interestingly, Gd-DTPA distribution was observed mostly in white matter, possibly due to a path of least resistance along the fibers of white matter tracts. Additionally, when the infusion was performed at sites of the cortical grey matter and white matter interface, Gd-DTPA distributed preferentially into white matter. This treatment was generally well tolerated and there was no surgery-related morbidity or mortality observed. Adverse events included worsening of pre-existing neurological symptoms, laboratory changes, and elevated body temperature, which were transient. Median survival was 28.1 ± 3 weeks (CI, 23.2–32.6) and median time to progression was 8 ± 6 weeks (CI, 6.8–9.2). More than 50% tumor reduction was observed in two out of eight treated patients. 

#### 2.5.2. Liposomal Irinotecan (Onivyde)

Kumar et al. presented interim results of a phase I study in which a liposomal preparation of irinotecan, an antineoplastic topoisomerase I inhibitor, was delivered via CED using real-time imaging [38]. At the time of presentation, this study enrolled 10 patients with recurrent high-grade glioma into two dosing cohorts to receive 20 or 40 mg/mL liposomal irinotecan with a co-infusion of gadolinium tracer. The volume of infusion was personalized to best cover each patient’s tumor and ranged from 2 to 17 mL. Seven patients lived over one year after treatment [38]. 

### 2.6. Oligodeoxynucleotides 

#### 2.6.1. CpG-28

CpG-28 is an oligodeoxynucleotide (ODN) containing CpG motifs. Human Toll-like receptor 9 is sensitive to unmethylated CpG motifs, since they are present at higher frequencies in bacterial DNA [40]. CpG-28 essentially acts as an analog to a bacterial pathogen-associated molecular pattern (PAMP) and interacts with TLR9 and subsequently stimulates an immune response [58]. A phase I study of intratumoral CpG-28 was conducted in 24 patients with recurrent glioblastoma. One to two catheters were placed intratumorally into contrast-enhancing areas and at least 2 cm from the surface of the brain. Patients were enrolled into six escalating dose levels from 0.5–20 mg [39]. A volume of 1 mL was delivered per catheter over the course of 6 h. One patient experienced a DLT (aphasia, hemiparesis, and visual disorders) which were not reversible. The authors note that this patient was deteriorating at the time of inclusion and it is unclear whether this was a treatment-related toxicity or due to disease progression. Three additional patients were treated at this dose level without any further DLTs. Other adverse events were generally mild and included lymphopenia, fever, fatigue, and worsening of previous neurological condition. Median survival was 7.2 months (95% CI, 4.8–12.7) and PFS6 was 4.5%. The authors recommended increasing the number of catheters in future trials to improve distributions; however, they note that complete tumor coverage is not a requisite for CpG to exert therapeutic effect. 

A subsequent phase II trial of CpG-28 was conducted in 31 patients with recurrent glioblastoma. In this trial, two catheters were placed intratumorally and 20 mg of CpG-28 was delivered at 4 mg/h over 6 h. The total volume infused was 2 mL. Twenty-two patients experienced transient worsening of baseline neurological symptoms, which were responsive to steroid therapy. Other adverse events included local infection, postprocedure pneumoencephaly, seizures, and a hemorrhage that led to death 8 days after catheter placement. Median survival was 28 weeks, PFS6 was 19%, and median PFS was 9.1 weeks. The targeted PFS benefit was not met [40]. 

#### 2.6.2. AP12009

AP12009 is an antisense ODN that binds to and silences TGFβ-2, which is over-expressed in glioma and drives the malignant phenotype [59]. AP12009 was explored clinically in three phase I/II studies, for which a pooled analysis is described in Hau et al., 2007. A total of 24 patients were treated across all three of these studies. In the third phase I/II study, AP 12009 was administered intratumorally over 4–7 days at a rate of 8 μL/min. Patients received at least two cycles of the study drug and offered additional subsequent cycles if clinically stable. Out of the 169 adverse events reported, 28 (16.6%) were thought to be possibly related to the study treatment and were described as central and peripheral nervous system disorders. One case of brain edema was considered a serious adverse event. No MTD was reached. Seven patients showed stable disease at 28 days. Overall survival was 44 weeks (range 18.9–87.9) for patients with glioblastoma and 146.6 weeks (range 32.0–276.3) for patients with anaplastic astrocytoma. 

### 2.7. Other Agents 

#### 131I-chTNT-1/B MAb (Cotara) 

131I-chTNT-1/B, a 131I-labeled chimeric monoclonal antibody that binds to histone H1, is a universal intracellular antigen that becomes exposed in the necrotic core of solid tumors. Once the antibody binds, 131I emits a cytotoxic dose of radiation to the surrounding tumor cells [41]. A total of 51 patients with malignant glioma were treated in initial phase I/II trials- 12 were enrolled in phase I and 39 into phase II. The first six patients were prescribed target volumes calculated to administer 137 Gy radiation. Subsequently, the dose was adjusted for tumor size and ranged from 0.5–3.0 mCi/cm^3^. Barium-impregnated cardiac/peritoneal catheter tips were placed at the center of the contrast enhancing tumor and 131I-chTNT-1/B Mab was infused in one to two infusions over a period of 1–2 days at 0.18 mL/h. Between 4.5 and 18 mL were infused. The most common adverse events were brain edema, hemiparesis, and headache. Procedure related events included post-procedural pain, catheter site pain, and catheter site redness/bleeding. One patient at the highest dose level died as a result of radiation-induced necrotic changes. Among recurrent glioblastoma patients receiving doses within the therapeutic window (*n* = 12), the median survival was 37.9 weeks (24.1–69.4). 

## 3. Limitations and Recent Developments in the Preclinical Setting 

In addition to the aforementioned issues of infusate reflux, suboptimal catheter placement, use of non-specialized catheters, and inability to trace drug substances, further limitations have been discovered as interest in CED has grown. Lack of standardization of infusion rates coupled with variance in duration of drug delivery between clinical trials may have contributed to the large volume of high-profile studies that have failed, impeding the clinical success and approval of CED for short- and long-term uses. Notable limitations unrelated to the delivery method include those pertaining to drug distribution and selection of appropriate therapeutic agents.

### 3.1. Drug Distribution

The pressure differential utilized in CED drives fluid into parenchymal tissue in a uniform manner. The raised interstitial fluid flow (IFF) may alter parenchymal morphology, therapeutic and molecular transport, extracellular matrix (ECM) arrangement, cellular motility, and ultimately may facilitate seeding of malignant cells to an unknown extent [21]. In particular, delivery within or near white matter (WM) tracts, ventricles, and ependymal surfaces due to raised IFF has been noted to cause CED failure due to either preferential flow away from the tumor and its surrounding parenchyma or by acting as a sink for infusate to collect. CED may also directly result in a necrotic core by depleting malignant cells, which can further act as a sink for therapeutic agents to statically collect, decreasing effective drug distribution [60]. In the setting of DIPG, it has also been postulated that the transverse and longitudinal fiber bundles in the brainstem may complicate infusate flow [46]. The complexity of such transport pathways has made it difficult to predict drug distribution from clinical magnetic resonance imaging (MRI) alone. Appreciating this, Jamal et al. investigated how hydraulic permeability of cerebral WM is affected by local micro-structural features such as axonal directionality and demonstrated quantitatively in a physical ovine brain model that hydraulic permeability is a function of WM fiber bundle direction [61]. 

Appreciating the anisotropic characteristics of brain parenchyma also remains important to understand how spatial distribution varies between therapeutic agents and how to boost treatment efficacy. This is largely crucial given that distribution within tumors has been proven in the clinical setting to be anisotropic, with infusate pooling in necrotic areas prior to efflux to the peritumoral region [54]. It was hypothesized that the differences with respect to peritumoral distribution could be largely attributed to the significant increase in IFF pressure and tissue heterogeneity within tumors [54]. Pertinent to this, Zhan et al. examined the response of various chemotherapeutic agents during CED simulated using a multi-physics model incorporating IFF, tissue deformation, and associated drug transport mechanisms [62]. Fluorouracil, carmustine, cisplatin, and doxorubicin could be delivered in large volumes when administered into relatively isotropic tissue, while paclitaxel and methotrexate were able to cover enlarged regions upon delivery into anisotropic tissues [62]. Similarly, Brady et al. modelled parenchymal fluid mechanics using simulations of intra-tumoral infusions in GBM patients, measuring distributions of traced compounds with MRI and positron emission topography (PET) [63]. From this, they confirmed that convection dominates over effects of diffusion in the context of biological agents or protein-sized molecules delivered by CED despite very small fluid speeds. In addition, they also determined that infusion-induced expansion of intercellular spaces and loss of small tracer molecules through a compromised BBB were key determinants of flow. The former phenomenon was responsible for the anisotropic distribution of therapeutic agents as opposed to cell geometry or directionality of neuronal pathways. Specifically, expansion of WM was found to significantly raise its hydraulic conductivity, making the tissue less anisotropic. This suggests that shunting of infusions in WM tracts is not primarily due to anisotropy of the neuronal fibers, but rather due to their expandability that permits conductivity to increase dramatically [63]. 

Others have reported the potential benefits of raised IFF in maintaining therapies within interstitial spaces where they move more slowly, so that they may access more invasive cells or exert effects for longer [21]. This hydraulic environment could also enhance convective drug transport and reduce fluid loss from the blood circulatory system to prevent drug concentration dilution [62]. The precise role of increased IFF thus needs further definition to permit appropriate use of CED and allow for case-by-case specialization. In light of this, Chatterjee et al. utilized dynamic contrast-enhanced MRI (DCE-MRI) using a para-magnetic contrast agent, e.g., gadolinium, to characterize IFF patterns within tumors and their surrounding parenchyma; they discovered a strong positive correlation between flow velocity magnitude and survival, which was not observed when analyzed against changes in diffusion. This may be due to improved drug transport within the tumor and towards the parenchyma, warranting further research combined with more advanced mathematical modeling to predict therapeutic response and disease progression [64]. To this end, Vidotto et al. proposed a novel computational model for predicting drug distribution during intra-cranial CED, integrating diffusion tensor imaging (DTI) and neurite orientation dispersion and density imaging (NODDI). The former non-invasively allows orientation of neural fibers to be determined as well as being able to differentiate between white and grey matter. However, DTI fails to capture information about the extracellular space volume fraction within brain parenchyma, which directly relates to hydraulic permeability. In light of this, NODDI allows for more accurate modelling of brain microstructures and intracellular, extracellular, and CSF compartments. Significant differences in drug distribution were observed using this two-pronged approach, with DTI-based models tending to overestimate drug distribution compared to the novel DTI-NODDI hybrid [65]. Regardless of predictive models, accurate real-time imaging of drug distribution during CED remains widely unavailable. Stephen et al. used real-time MRI in murine GBM models to investigate the dispersion of infusate by CED using iron oxide nanoparticles (NP) targeting GBM ligand CTX. These functionalized NP successfully targeted GBM cells, localized to nuclei, and provided an accurate means for the real-time monitoring of infusate that remains non-existent in clinical practice [66].

### 3.2. Hardware, Infusion Technique, and Long-Term Convection Enhanced Delivery 

To optimize intra- and peri-tumoral drug distribution, emphasis on cannula design has risen over the years, with modifications such as porous-tipped catheters for improved uniformity of distribution and balloon-tipped cannulas to increase the surface area in contact with malignant tissue [17,23,67,68,69]. Attempts to address the well-documented issue of infusate reflux have been made, such as by using stepped catheters, but most studies fail to mention or estimate the effects of said backflow [70]. To assess this, Orozco et al. proposed a novel 3D non-linear biphasic finite element model of a healthy human brain to quantify the extent of reflux during CED, where cannula diameter and placement could be adjusted. This model was able to predict the length of backflow, accounting for both patient-specific structural factors and the non-linear deformation of brain parenchyma [71]. 

Taking this further, needle tip structure was investigated to determine how to reduce infusate reflux during CED. Sharp and blunt tips were compared under different flow rates in an agarose hydrogel brain phantom; sharp needles were associated with significantly greater backflow lengths at higher flow rates, but no significant differences were noted between tip geometry at lower flow rates. This suggests that sharp tips limit the higher flow rates that are typically required to deliver larger drug volumes over shorter periods of time [72]. Mehta et al. also used agarose gel brain phantoms to evaluate how controlled catheter movements during CED impact dye dispersal. A single-port stepped catheter was used to study stationary infusion, continuous retraction, continuous insertion, and intermittent insertion during infusion. The continuous retraction and intermittent insertion protocols showed significantly higher volumes of distribution than traditional stationary catheters, which were found to have greater backflow and lower forward flow distances. This highlights how tumor coverage can be improved by using dynamic catheters [73]. Apart from infusate reflux, cerebral catheters can also become obstructed during chronic CED. Quintero et al. proposed various surgical techniques to avoid this, including a hollow intra-luminal stylet to maintain catheter rigidity that can be infused with saline during catheter insertion [74].

Poor understanding of intra-tumoral drug distribution and optimal catheter placement have led to inconsistent infusion protocols between CED studies [70]. Insertion of multiple catheters has been trialed to better distribute and increase overall infusion at lower rates but with limited success due to surgical access [23,75]. Others have used robot-guided infusions to optimize placement with varying success [23]. In light of this issue, computational optimization methods for catheter insertion and maximal drug distribution in CED have been proposed, accounting for diffusion, viscous resistance, fluid pressure, tumor geometry, and porosity [76]. Further studies highlighted the use of patient-specific diffusivity and permeability parameters in computational fluid dynamics simulations to better guide catheter placement in CED [77]. Infusion in CED is classically pressure-driven, but this approach has been limited by poor control of infusate flow due to the pressure gradient causing backflow along the cannula or across ependymal linings. Faraji et al. proposed a novel infusion approach called electrokinetic CED (ECED), which induces fluid flow using an electrical current passed through a conducting solution surrounded by charged walls. Thus, the infusion path is determined by current density and tissue resistance. Fused silica cannulas were used to permit current and fluid flow, with electrodes supporting fluid flow in vessels outside the brain. This novel technique was capable of delivering therapeutic agents to local parenchymal areas with greater control and lower pressure thresholds than traditional CED [78]. 

Infusion remains limited to short-term use by current ports and external catheters, which fail to facilitate long-term delivery indicated in chemotherapy regimens for optimal efficacy. Thus, specific tumor-targeting strategies and modifications for chronic delivery are being considered. Specialized pumps, distribution prediction software, and MRI-guided techniques are necessary to optimize the treatment of larger tumor volumes in the future. To this effect, D’Amico et al. described a fully internalized pump-catheter system for chronic CED in a porcine animal model, which was validated as safe and well-tolerated at high flow rates. Gadolinium contrast was used as a surrogate for drug distribution and is known to be rapidly cleared in animal studies, emphasizing the need for long-term infusions [79]. Co-infused topotecan and gadolinium alongside MRI were used to assess the volume of drug distribution, which remained large and stable for up to 32 days using this implantable pump-infusion system [80]. Besides via a single, continuous prolonged infusion, chronic CED may alternatively be delivered via repeat infusions. Sequential infusions reported in a pediatric population of diffuse intrinsic pontine glioma (DIPG) patients showed that past infusions did not negatively influence future infusions, catheter insertion accuracy, or distribution capacity [81]. This work verifies the safety of repeat CED in a pediatric cohort and warrants similar study in adults. 

Optimization of the duration of infusion in chronic CED has also been poorly studied, with the length of infusion varying widely between different trial protocols. Drug molecular weight may be an important consideration and pre-clinical models of DIPG have shown that low molecular weight agents are cleared more rapidly and may benefit from prolonged infusions as a result [82]. Lewis et al. reviewed current catheters available to guide development of chronic CED [60]. Those that become flexible after insertion are being considered for mobile patients along with cannulas made of biocompatible materials, e.g., carbothane, for chronic insertion without notable reflux or tissue adherence [23]. Best practice may also include catheter placement around the periphery of malignant tissue, especially if there is a high risk of recurrence [60]. Initial characterization of drug distribution followed by periodic monitoring is necessary to assess for changes in distribution in long-term CED. Flow imaging may offer a different perspective on treatment outcomes that would not be discernable from imaging of the tumor and surrounding parenchyma alone. However, further definition of the roles of tissue components such as ECM and microvasculature in the context of CED is required to examine drug transport properties and improve drug distribution [83]. 

### 3.3. Therapeutic Agents

The effects of therapeutics on CED efficacy have been extensively reviewed elsewhere [23]. Despite direct, intra-tumoral delivery offered by CED, therapy must also target infiltrative malignant cells within peri-tumoral WM. Agents delivered by CED should ideally have a wide therapeutic index and toxicities limited to tumor cells, contraindicating agents that are excessively toxic to healthy WM [23]. Traditional chemotherapy drugs produced prior to the inception of direct delivery were modified to increase lipophilicity and decrease molecular weight to improve transport through vasculature and the BBB. However, if delivered by CED, drug distribution may be hindered by these properties and may benefit from re-modification, although these agents are still being tested using CED [84,85].

Smaller agents and NP may utilize the transient edema created by infusions that expand the extracellular space to improve tumor penetration and distribution. However, smaller agents have also been noted to be rapidly metabolized or eliminated, thus failing to distribute effectively [23]. Jahangiri et al. postulated that this would require more precise control of the volume of delivery, which could lead to a decrease in efficiency and clinical application of CED [23]. NP avoid rapid clearance but tend to remain immobilized within the extracellular space at the point of injection, most likely due to the nanoporous and adhesive nature of ECM [86]. NP drug carriers in CED are novel in their abilities to be delivered in a controllable manner and sustain drug release over time [86]. Regarding the issue of immobilization at the administration site, Zhang et al. noted that dense polyethylene glycol (PEG) corona on NP removed adhesive trapping in brain parenchyma. Rapid brain tissue penetration in rats was demonstrated with brain-penetrating NP (BPN) < 114 nm in diameter in healthy tissue and <70 nm in malignant tissue. BPN achieved uniform, widespread distribution, sustaining delivery of therapeutic agents without the significant acute toxicities associated with dose-matched free agents. Thus, CED led to a greater homogenous volume of distribution within the site of administration (i.e., striatum) and decreased presence in WM tracts compared to manual injection [86]. 

Liposomal drug carriers have similarly been demonstrated to improve homogenous drug distribution, drug accumulation, and penetration into large volume tumors when infused by CED rather than systemically due to the enhanced tumor cell specificity and lower elimination rates of these formulations. Variation in parameters such as liposome solution concentration, surface receptor modulation, and vascular permeability may further improve therapeutic efficacy. Shi et al. compared liposomal formulations of chemotherapeutic agents such as oxaliplatin to standard preparations when delivered by CED in F98 glioma-bearing Fischer rats. Liposomal encapsulation was found to triple the maximum tolerated dose of therapeutic agent delivered by CED and increase intra-tumoral drug distribution while maintaining a similar median survival time and anti-tumor potential to standard formulations [87]. Noting the reduced toxicity and enhanced distribution of liposomal carriers, Shi et al. went on to investigate the effects of pegylation and charge of liposomal cisplatin formulations in the same rat model as well as in vitro. Pegylated carriers were found to travel longer distances when delivered by CED, irrespective of charge status, thus improving overall drug distribution. However, discrepancies were interestingly noted between the in vitro and in vivo therapeutic effects of anionic versus cationic liposomes. The anionic, pegylated formulation yielded the longest median survival in the F98 glioma-bearing Fischer rat model, but was less efficient in vitro compared to other formulations [88]. Cationic liposomes conversely demonstrated the highest therapeutic efficacies in vitro due to more efficient cell membrane binding and enhanced carboplatin accumulation in tumor cells, but this property failed to significantly improve median survival time in vivo. Han et al. similarly demonstrated that pegylated liposomal carriers modified with cationic lipids exhibited specific uptake by GBM cells in vitro, but also showed efficient distribution and retention in vivo in malignant mouse brain models post-CED [89]. These inconsistent findings emphasize the need to consider relevant animal GBM models to be able to accurately comment on the optimal biochemical characteristics of liposomal drug carriers delivered by CED.

CED has also been considered for delivery of sensitizing agents, photodynamic therapy, sonodynamic therapy, neutron capture therapy, and radiotherapy in the preclinical setting. This is important especially in the case of binary approaches such as those highlighted by Elleaume et al., in which they demonstrated that the therapeutic efficacy of cisplatin and irradiation was enhanced given their delivery using CED [90].

CED may also diminish the expected impact of reduced diffusion with larger liposomal drug carriers on drug delivery, as convective forces tend to dominate over diffusive transport, warranting further research into liposomal carriers of varying sizes. Zhan et al. showcased that penetration of therapeutic agents into deep tumor regions was prolonged for the first 12 h after CED infusion, while its concentration in healthy tissue remained low for the first 6 h. Thus, CED in conjunction with liposome-encapsulated agents allowed for more effective treatment in less vascularized tumors, owing to the reduced exchange surface area within the tumor and reduced fluid loss that further decreased elimination rates [60]. In light of this, Zhan et al. later investigated the use of bevacizumab combined with cytotoxic agents administered by CED using a 3D realistic brain tumor model. These multi-physics simulations showed that the volume of drug distribution was improved most significantly when bevacizumab was combined with doxorubicin, owing to the significant reduction in fluid loss from the blood circulatory system and inhibition of therapeutic dilution [83].

## 4. Discussion

CED is an attractive choice for drug delivery into the CNS due to the possibility of achieving high doses of therapeutics in the target area while limiting systemic toxicity. While the technique, devices, and parameters are still being optimized, early trials demonstrated that this method is safe and can significantly improve locoregional delivery over diffusion-based approaches. The development and optimization of CED poses several challenges. Perhaps the most widely encountered challenge, as evidenced by prior clinical trials, is elucidating optimal catheter positioning to achieve adequate distribution within the targeted region of brain. Chen et al., 1999, cautioned that infusate can reflux along the catheter–brain interface [91]. This is possibly a reason for the failure to generate uniform Vd in many instances where “off-the-shelf” catheters were used. Backflow and subsequent leakage of infusate out of the burr hole used to place the catheter was observed by both Popperl et al. and Tanner et al., which further raises concern around this phenomenon [24,25]. 

Utilization of an optimized catheter is critical to conducting trials with CED, as inefficient distribution can negatively confound efficacy endpoints of therapeutics that may, in fact, be good candidates for treatment. As noted by Sampson et al., 2010, the phase III PRECISE trial may have failed to meet its efficacy endpoint due to ineffective delivery [53]. Clinical trials are a labor- and resource- intensive process and it may be very unlikely to secure the funding and resources to repeat clinical trials in agents that have already produced suboptimal results, even if these results may be due to delivery. Although the design and production of catheters that are specific for CED come with regulatory challenges, it is necessary to optimize delivery. 

Another challenge faced in CED trials is the need to separately assess the efficacy of drug candidates and the efficiency of drug delivery. Tissue biopsy with a validated biomarker is the gold standard for confirming drug delivery to the targeted region of brain; however, this is invasive and accurate surrogates for Vd should be investigated. Current approaches include co-infusion of drug with a tracer molecule (which may introduce regulatory challenges, as this combination could be considered a new drug entirely), radiolabeling the drug molecule itself with a traceable element (which may affect the efficacy of the drug), or conducting a “pre-test” infusion with a tracer alone [37,55]. Using a tracer molecule as a surrogate for drug distribution raises questions regarding accuracy, as molecules must be matched in size, hydrophilicity, and charge, as all of these factors affect distribution in the brain. Additionally, the possibility that the drug and tracer molecule are bound differently by efflux pumps could also create a discrepancy between the perceived and true distribution. The approach explored by Sampson et al., 2007, in which software was used to retroactively predict drug distribution based on catheter trajectory holds great potential for a non-invasive and non-toxic predictor of Vd if it can be further developed to improve sensitivity. 

Perhaps the most ubiquitously encountered challenge was the seeming lack of predictability in the behavior of infusate. Findings from multiple trials shape future recommendations for optimizing delivery in CED trials, which are summarized in Table 2. Voges et al. found that infusate more readily distributed into white matter tracts rather than grey matter, which was possibly due to a path of least resistance that existed along the fibers of white matter tracts. Additionally, Vd was higher in areas of tumor-infiltrated brain and normal brain parenchyma when compared with areas within tumor. These findings cumulatively suggest that areas of the brain along the interface of white and grey matter or within tumors are not ideal targets for CED. Additionally, intratumoral infusions carry greater risk for complications due to mass effect, both due to the additional volume of infusate and due to secondary edema, which occurs with the delivery of certain therapeutic agents. Patients with resectable tumors may be a more ideal target population for CED treatment, as tumors can be removed and reduce the risk for mass effect and the surrounding tumor infiltrated brain can be more uniformly targeted by infusion. Of course, glioma treatment is a dynamic process, as changes occur in tumor density, development of necrosis, edema, neovascularity, and many other variables that will require the ability to constantly fine-tune CED-directed therapies. Nonetheless, emerging clinical trials are taking these variables into account in addition to incorporating innovative catheter designs, multiport catheters, subcutaneous implantable pump systems for chronic drug delivery, therapeutic designs tailored towards convection-enhanced delivery and many more considerations (Table 2).

As noted by Kunwar et al., 2007, it is critical to consider the timing at which catheter placement is performed [51]. If done at the same time as resection, significant brain shift and re-expansion can occur, which would reduce the accuracy of pre-operative MRI. Although a separate operative procedure to place catheters may be less favorable due to cost and extended length of hospital stay, it may improve the accuracy of catheter placement and ultimately reduce the likelihood of ineffective treatment due to backflow and leakage into the CSF compartment.

## 5. Future Directions

The most glaring limitation of CED is the procedural burden of re-dosing patients who are stable or respond favorably to therapy. The use of a catheter and syringe pump may require multiple surgical procedures to repeat cycles of treatments. Catheters have been left in place for several days at a time. While this offers some improvement to therapeutic delivery, implantable devices that can sustain long-term repeated cycles of therapy are likely the superior alternative; however, they may be ineffective for gliomas that are progressive at distant brain sites [82,93]. 

Optimization of CED catheters, pumps, and tracers will allow for better characterization of infusion parameters and allow for more targeted and reliable delivery of a variety of therapeutics into the CNS.

## Figures and Tables

**Table 1:** Completed clinical trials utilizing CED to deliver similar agents.

Reference	Therapeutic Agent	Population	Dose	N	Catheter	Catheter Size	Infusion Rate	Volume Infused	Duration	Adverse Events	Outcome
Lidar et al. [23]	Paclitaxel	Recurrent malignant glioma	0.55, 1.2 mg/mL	15	Medtronic VP shunt catheter	-	0.3 mL/h	6–6.6 mL/day	2–5 days	Chemical meningitis	Median survival = 7.5 months
Tanner et al. [24]	Paclitaxel	rGBM	0.5, 0.25 mg/mL	8	Medtronic VP shunt catheter	1.5 mm inner, 3 mm outer diameter	0.3 mL/h	26 mL	120 h	Edema, neurological deterioration, skin necrosis	Survival 4–15 months
Popperl et al. [25]	Paclitaxel	rGBM	0.5, 0.25 mg/mL	8	Silicone catheter	-	0.3 mL/h	-	120 h	Edema, skin necrosis	Survival 5–16 months
Bruce et al. [26]	Topotecan	Recurrent malignant glioma	0.02–0.133 mg/mL	16	2 Silastic CSF-peritoneal catheters	2.5 mm outer diameter	200 μL/h	40 mL	100 h	Parietal syndrome, dysmetria, hemineglect, weakness	Median survival = 60 weeks, PFS6 = 44%, mPFS = 23 weeks
Vogelbaum et al. [27]	Topotecan	Recurrent HGG	0.0067 mg/mL	3	2 Cleveland Multiport catheters	2.5 mm central shaft, 0.38 mm outer diameter per micro-catheter	0.396 mL/h (6.6 μL/min)	38 mL	96 h	-	-
Laske et al. [28]	Tf-CRM107	Recurrent malignant brain tumors	0.1–3.2 μg/mL	18	1–3 CSF-peritoneal catheters	2.5 mm outer diameter	0.5–10 μL/min	5–180 mL	2–16 days	Edema, seizures	Decrease in tumor volume, 13% complete response
Weaver and Laske [29]	Tf-CRM107	rGBM, AA	0.67 μg/mL	44	2 catheters	2.5 mm outer diameter	Up to 0.2 mL/h	40 mL	4–5 days	Edema, seizures	Median survival = 37 weeks
Weber et al. [30]	NBI-3001	Recurrent malignant glioma	6–15 μg/mL	31	Up to 3 infusion catheters	-	-	40 mL, 100 mL	96 ±4 h	Seizures, headache, weakness, edema	Median survival = 8.2 months, 5.8 for glioblastoma
Sampson et al. [31]	TP-38	Recurrent malignant brain tumors	25–100 ng/mL	20	Barium-impregnated CSF-ventricular catheters (2)	Outer diameter 2.1 mm	0.4 mL/h	40 mL	50 h	Hemiparesis, fatigue, subdural hygroma, hemorrhage	Median survival = 28 weeks
Vogelbaum et al. [32]	IL13-PE38QQR ^†^	Newly diagnosed malignant glioma	0.25 ug/mL, 0.5 μg/mL	22	Barium-impregnated silicone catheters (2–4)	1mm inner, 2 mm outer diameter	0.75 mL/h *	-	96 h	Seizure, aphasia, confusion, fatigue, gait disturbance, nystagmus	Survival range 5–113 weeks, PFS range 5–78+ weeks
Kunwar et al. [33,34]	IL13-PE38QQR	Recurrent malignant glioma	IT:0.25–2 μg/mLIP: 0.25–1 μg/mL	51	Barium-impregnated silicone catheters	Inner diameter 1–1.3 mm, outer diameter 2–2.5 mm	IT: 0.4 or 0.54 mL/h * IP: 0.75 mL/h *	IT: 19.2–51.8 mL IP: 72–108 mL	IT: 48–96 h IP: 96 h–6 days	Hemiparesis, convulsions, headache	Median survival = 45.9 weeks (95% CI, 37.4–59.3)
Kunwar [34]	IL13-PE38QQR	rGBM	0.5 μg/mL	183	Barium-impregnated silicone catheters	-	0.75 mL/h	-	96 h	Pulmonary embolism	Median survival = 36.4 weeks
Kicielinski et al. [35]	Reovirus	Recurrent malignant glioma	10^8^–10^10^ TIDC	15	1–4 Phoenix Biomedical CED catheters	-	400 μL/hour *		72 h	Catheter clogging, convulsions	Median survival = 140 days, mTTP = 61 days
Desjardins et al. [36]	PVSRIPO	rGBM	10^7^–10^10^ TIDC	61	1 Vygon catheter	-	500 μL/h	3.25 mL	6.5 h	Hemorrhage, headache, hemiparesis, seizure	Median survival = 12.5 months
Voges [37]	LIPO-HSV-1-*tk*	rGBM	-	8	1–2 silicon catheters	-	0.025–0.6 mL/h	3.5 mL	2 h	Transient worsening of neurological condition, elevated body temperature	Median survival = 28.1 ± 3 weeks, Median TTP = 8 ± 6 weeks
Kumar et al. [38]	Liposomal irinotecan (Onivyde)	Recurrent HGG	20, 40 mg/mL	10	Up to 4 catheters	-	-	2–17 mL	<5 h	-	Survival >1 year in 7 patients
Carpentier et al. [39]	CpG-28	rGBM	0.5–20 mg	24	1–2 catheters	-	0.2 mL/h	1 mL	6 h	Lymphopenia, worsening of neurological condition	Median survival = 7.2 months, PFS6 = 4.5%
Carpentier et al. [40]	CpG-28	rGBM	20 mg	31	2 catheters	-	4 mg/h	2 mL *	6 h	Transient neurological worsening, fever, hemorrhage at catheter site SAEs- infection, penumoencephaly, hemorrhage	PFS6 = 19% mPFS = 9.1 weeks Median survival = 28 weeks
Hau et al. [15]	AP-12009	Recurrent HGG	2.5–80 μM	24	1 catheter	-	4–8 μL/min	23.04–80.6 mL	4 days, 7 days	Brain edema	Overall survival = GBM: 44 weeksAA:146.6 weeks
Patel et al. [41]	Cotara	Malignant glioma	0.5–3 mCi ^131^I/cm^3^, 1 mg/mL	51	Barium-impregnated cardiac/peritoneal catheters	-	0.18 mL/h	4.5–18 mL	1–2 days	Edema, hemiparesis, headache	Median survival (GBM subset, *n* = 12): 37.9 weeks

* Denotes total infusion rate, which was divided by the number of catheters placed. ^†^ IL13-PE38QQR commercially known as citredekin besudotox.

**Table 2:** Ongoing clinical trials utilizing convection enhanced delivery.

Trial Registration	Therapeutic Agent	Phase	N	Status	Estimated Completion	Population	Study Design	Endpoints
NCT04608812 [92] *	OS2966	I	24	Active, recruiting	March 2022	Recurrent/progressive HGG	Treat-resect-treat (IT and IP infusions), Gadoteridol co-infusion	Safety (AEs, DLTs), optimal dose Distribution of infusion, tumor response rate, TTP
NCT01491893	PVSRIPO	I	61	Active, not recruiting	June 2021	Grade IV malignant glioma	IT infusion	MTD (DLTs), median OS, median PFS
NCT04479241 (LUMINOS-101)	PVSRIPO, pembrolizumab	II	30	Active, recruiting	March 2023	Recurrent glioblastoma	IT infusion	Objective response rate, duration of response, durable radiographic response, AEs, biomarker identification
NCT02303678	D2C7-IT	I	115	Active, recruiting	December 2022	Recurrent malignant glioma	IT infusion	MTD (DLTs), OS, association between EGFRvIII and EGFRwt expression and PFS/OS
NCT04547777	D2C7-IT, 2141-V11	I	30	Active, not yet recruiting	December 2025	Recurrent HGG	IT infusion	Safety (AEs, DLTs)
NCT04160494	D2C7-IT With Atezolizumab	I	18	Active, recruiting	January 2026	Recurrent grade IV glioma	IT infusion	Safety (AEs, DLTs)
NCT03927274	Topotecan	I	5	Active, recruiting	April 2022	Recurrent/progressive HGG	IT, Gd-DTPAco-infusion	Drug distribution, AEs, extent of backflow
NCT03283631	EGFR-vIII CAR-T Cells	I	24	Suspended pending protocol amendment	December 2022	Recurrent glioblastoma	IT infusion	MTD, change in T cell trafficking in tumor and systemically, median survival, median PFS
NCT03154996	Topotecan	I	5	Active, not recruiting	September 2021	Recurrent HGG	Chronic IT and IP infusion for 32 days via subcutaneous pump	Clinical toxicity rate, tumor response, PFS
NCT02869243	hrBMP4	Ib	15	Active, not recruiting	March 2021	Progressive/multiple recurrent glioblastoma	Treat-resect-treat (IT and IP infusions), Gd-DTPA co-infusion	MTD (DLTs), tumor response, distribution
NCT02022644	Liposomal irinotecan	I	18	Active, not recruiting	November 2021	Recurrent HGG	IT, gadolinium co-infusion	MTD (DLTs), tumor response rate, TTP, OS, infusion modeling and imaging, Vd/Vi, tumor histology
NCT01906385	186RNL (ReSPECT)	I/II	55	Active, recruiting	January 2025	Recurrent glioma	IT	MTD (DLTs), dose distribution on SPECT imaging, response rate, survival

* The authors disclosed a conflict of interest with the sponsor of this trial.

## Abbreviations

BBB	blood–brain barrier
Vd	volume of distribution
IFF	interstitial fluid flow
ECM	extracellular matrix
WM	white matter
OS	overall survival
PFS	progression-free survival
MTD	maximum tolerated dose
DLT	dose-limiting toxicity
DWI	diffusion-weighted imaging
FET	*O*-(2-[18F]fluoroethyl)-l-tyrosine
FDG	fluorodeoxyglucose
PET	positron emission tomography
CSF	cerebrospinal fluid
TGF-α	transforming growth factor-alpha
SPECT	single photon emission computed tomography
HAS	human serum albumin
TCID_50_	tissue culture infectious dose 50
ODN	oligodeoxynucleotide
TGFβ-2	transforming growth factor beta-2
Tf-CRM-107	transferrin receptor-based diphtheria toxin
NBI-3001	IL4-*Pseudoamonas* exotoxin
LIPO-HSV-1-*tk*	HSV-1-*tk* gene-bearing cationic liposomal vector
TCID50	50% tissue-culture infectious doses
^131^I-chTNT-1/BMAb	cotara-^131^I-labeled chimeric monoclonal antibody
TP-38	recombinant toxin targeting epidermal growth factor receptor (EGFR)
CpG-ODN	oligodeoxynucleotides containing CpG motifs
Delta-24-RGD	conditionally replication-competent adenovirus
Gd-DTPA	gadolinium-diethylene-triamine-pentaacetic acid
MRI	magnetic resonance imaging
PET	positron emission tomography
SPECT	single photon emission computed tomography
IT	intratumoral
IP	intraparenchymal
PFS6	progression free survival at 6 months
mPFS	median progression free survival
rGBM	recurrent glioblastoma
HGG	high-grade glioma
HGG	high-grade glioma
IT	intratumoral
IP	intraparenchymal
AE	adverse event
DLT	dose limiting toxicity
TTP	time to progression
PFS	progression free survival
OS	overall survival
Vd	volume of distribution
Vi	volume infused
Agents:	
OS2966	anti-CD29 mAb
PVSRIPO	polio-rhinovirus recombinant oncolytic virus
D2C7-IT	recombinant immunotoxin targeting EGFRwt and EGFRvIII
2141-V11	anti-CD40 mAb
Gd-DTPA	gadopentetic acid
EGFR-vIII CAR	chimeric antigen receptor (CAR) that recognizes epidermal growth factor receptor variant III
hrBMP4	human-recombinant bone morphogenetic protein 4
